# Postremission sequential monitoring of minimal residual disease by WT1 Q‐PCR and multiparametric flow cytometry assessment predicts relapse and may help to address risk‐adapted therapy in acute myeloid leukemia patients

**DOI:** 10.1002/cam4.593

**Published:** 2015-12-29

**Authors:** Michele Malagola, Cristina Skert, Erika Borlenghi, Marco Chiarini, Chiara Cattaneo, Enrico Morello, Valeria Cancelli, Federica Cattina, Elisa Cerqui, Chiara Pagani, Angela Passi, Rossella Ribolla, Simona Bernardi, Viviana Giustini, Cinzia Lamorgese, Giuseppina Ruggeri, Luisa Imberti, Luigi Caimi, Domenico Russo, Giuseppe Rossi

**Affiliations:** ^1^ Unit of Blood Disease and Stem Cell TransplantationDepartment of Clinical and Experimental SciencesUniversity of BresciaAO Spedali Civili di BresciaBresciaItaly; ^2^ Division of HematologyAO Spedali Civili di BresciaBresciaItaly; ^3^ Centro di Ricerca Emato‐oncologica AIL (CREA) BresciaAO Spedali Civili di BresciaBresciaItaly; ^4^ Laboratorio AnalisiDepartment of Molecular and Translational MedicinUniversity of BresciaAO Spedali Civili di BresciaBresciaItaly

**Keywords:** LAIP, minimal residual disease, WT1

## Abstract

Risk stratification in acute myeloid leukemia (AML) patients using prognostic parameters at diagnosis is effective, but may be significantly improved by the use of on treatment parameters which better define the actual sensitivity to therapy in the single patient. Minimal residual disease (MRD) monitoring has been demonstrated crucial for the identification of AML patients at high risk of relapse, but the best method and timing of MRD detection are still discussed. Thus, we retrospectively analyzed 104 newly diagnosed AML patients, consecutively treated and monitored by quantitative polymerase chain reactions (Q‐PCR) on WT1 and by multiparametric flow cytometry (MFC) on leukemia‐associated immunophenotypes (LAIPs) at baseline, after induction, after 1st consolidation and after 1st intensification. By multivariate analysis, the factors independently associated with adverse relapse‐free survival (RFS) were: bone marrow (BM)‐WT1 ≥ 121/10^4^ ABL copies (*P* = 0.02) and LAIP ≥ 0.2% (*P* = 0.0001) (after 1st consolidation) (RFS at the median follow up of 12.5 months: 51% vs. 82% [*P* < 0.0001] and 57% vs. 81%, respectively [*P* = 0.0003]) and PB‐WT1 ≥ 16/10^4^ ABL copies (*P* = 0.0001) (after 1st intensification) (RFS 43% vs. 95% [*P* < 0.0001]) Our data confirm the benefits of sequential MRD monitoring with both Q‐PCR and MFC. If confirmed by further prospective trials, they may significantly improve the possibility of a risk‐adapted, postinduction therapy of AML.

## Introduction

During the last decades, the treatment of acute myeloid leukemia (AML) has not significantly changed and the outcome has remained largely unsatisfactory 1, 2. Complete remission (CR) can be achieved in 70–80% of newly diagnosed AML patients treated with conventional induction/consolidation regimens, but relapses still occur in 40–50% of cases and, in the end, no more than 30–40% of adult AMLs can be cured 2, 3. Therefore, the optimization of postremission therapy to maintain CR represents the greatest challenge in AML treatment.

Thanks to a wider use of allogeneic stem cell transplantation (allo‐SCT), which is the most powerful postremission treatment 2, some advances have been registered in younger adults with high‐risk disease. Nowadays, there is general agreement in offering allo‐SCT in first remission to AML patients falling into the category of unfavorable cytogenetics 4. They account for 15–20% of newly diagnosed patients and <5–10% of them may become long survivors without allo‐SCT 4. Excluding another 15–20% of patients with favorable cytogenetics, who can be cured in up to 60–70% of cases without allo‐SCT 2, in the remaining 40–50% of patients with intermediate cytogenetic risk, the therapeutic decision is problematic due to their heterogeneity and to the difficulty in precisely defining their prognosis. Different clinical (e.g., age, secondary AML, extramedullary involvement) and laboratory (e.g., white blood cell count, LDH serum levels) factors have been identified and correlated with prognosis 2, but none of them, neither alone nor in combination, has been universally recognized and systematically applied to guide risk‐adapted therapeutic strategy.

More recently, advances in defining the prognostic relevance of genomic alterations created an enormous expectation in understanding the biological heterogeneity of AML and in guiding the therapy 5. Unfortunately, due to the variety and infrequency of molecular abnormalities, the translation of this genomic information into clinic is difficult and, as a consequence, AML therapeutic strategy based on the molecular data at diagnosis still remains controversial for the great majority of patients.

Data reported by the Medical Research Council (MRC) and Gruppo Italiano Malattie Ematologiche dell'Adulto (GIMEMA) strongly suggest that the use of posttreatment factors indicating the speed and quality of response can improve the outcome prediction in AML patients 6, 7, 8, 9, 10, 11, 12. The MRC study showed that patients who entered CR after the 2nd induction course had a worse outcome 6, whereas GIMEMA studies showed that assessment of the minimal residual disease (MRD) might be a powerful and accurate tool to improve risk evaluation, as initially established on the basis of cytogenetic and molecular markers 8. In this view, multiparametric flow cytometry (MFC) and quantitative polymerase chain reactions (Q‐PCR) on target genes, such as the WT1 pan‐leukemic marker, are the techniques which are currently used to evaluate the quality of response after chemotherapy‐induced morphological CR 7, 9. At present, the predictive power of each technique and the identification of the most accurate time‐point for MRD assessment are not well defined and still remain open questions. Furthermore, it is a matter of debate how and when MRD data should be used in the context of the AML treatment program.

The aim of this study was to comparatively analyze the predictive impact of sequential MRD monitoring with leukemia‐associated immunophenotypes (LAIP)‐MFC and WT1 Q‐PCR in a cohort of 104 consecutively treated AML patients.

## Patients and Methods

### Patients

One hundred and four consecutive AML patients admitted to the Haematology Department of Spedali Civili in Brescia from 2010 to 2013 and consecutively treated with a conventional induction/consolidation treatment program 13 were retrospectively analyzed. The clinical and biological features of the patients are reported in Table 1. According to cytogenetic and molecular European Leukemia Net (ELN) criteria 1, 37/104 (36%), 30/104 (29%), 17/104 (16%), and 20/104 (19%) patients belonged to the low, intermediate‐1, intermediate‐2, and high‐risk categories, respectively; thus, 65% of the cases fell in the favorable/intermediate‐1 risk ELN categories. At diagnosis, a LAIP‐MFC was available in 80/104 (77%) cases, whereas bone marrow (BM) and peripheral blood (PB) WT1 Q‐PCR was available in 92/104 (88%) and 70/104 (67%) cases, respectively. Overall, in 46 cases from BM (*n* = 12) and PB (*n* = 34) WT1 levels were not assessable due to lack of sample collection (92% of the cases) or technical reasons (8% of the cases). Nevertheless, all the patients had at least one sample, either from BM or PB, available for WT1 quantitative assessment, and WT1 was always overexpressed in these samples. 21% of patients were positive for Flt3 internal tandem duplication, 42% for the NPM1 mutation, and 14% for both. The median follow up of the study population is 12.5 months (range: 1–47).

**Table 1:** Clinical and biological features of 104 AML patients

	*N* (%)
Median age (range)	59 (18–75)
Sex (*male*)	59 (57)
Median WBC count/*μ*L (range)	9715 (400–218,700)
Unfavorable cytogenetic^b^	44 (43)
ELN risk category^a^	
Favorable	37 (36)
Intermediate‐1	30 (29)
Intermediate‐2	17 (16)
High‐risk	20 (19)
LAIP at diagnosis	80 (77)
BM‐WT1 available at diagnosis^b^	92 (88)
PB‐WT available at diagnosis^b^	70 (67)
FLT3 ITD mutation	22 (21)
FLT3 TKD mutation	10 (10)
NPM1 mutation (on 99 patients)	42 (42)
NPM1/FLT3ITD mutations (on 100 patients)	14 (14)
Patients addressed to consolidation	93 (83)
Patients addressed to intensification	80 (77)
Patients addressed to allo‐SCT	33 (32)

LAIP, leukemia‐associated immunophenotype; ITD, internal tandem duplication; PBSC, peripheral blood stem cells.

a According to the ELN criteria 1.

b According to the ELN criteria 15.

### Plan of treatment

All the patients, stratified according to the ELN risk 1, received a treatment program according to the NILG (Northern Italy Leukemia Group) AML‐Protocol 13.

Briefly, patients less than 70 years received an induction chemotherapy consisting of ICE regimen (idarubicine 12 mg/m^2^ per day on days 1, 2, and 3; etoposide 100 mg/m^2^ per day on days 1–5; cytarabine 100 mg/sqm bid on days 1–7), followed by SPLIT regimen (idarubicine 17.5 mg/m^2^ per day on days 1 and 8, cytarabine 3 g/sqm bid on days 2, 3, 9, 10) in case of no response (NR) to ICE; patients older than 70 years were treated according to the MICE regimen (mitoxantrone 7 mg/m^2^ per day on days 1, 3, 5, cytarabine 100 mg/m^2^ per day continuous infusion on days 1–7, etoposide 100 mg/m^2^ per day on days 1, 2, 3). Consolidation treatment included one cycle of idarubicine 10 mg/m^2^ per day on days 1, 2, and 3 and cytarabine 200 mg/m^2^ per day on days 1–7, followed by one cycle of intermediate‐dose cytarabine (2 g/sqm per day for 4 days) for patients younger than 70 years and one cycle of mini‐ICE regimen followed by one cycle of intermediate‐dose cytarabine (2 g/sqm per day for 3 days) for patients older than 70 years. Both consolidation programs were followed by peripheral blood stem cell (PBSC) collection. Patients with low and intermediate risk leukemia were addressed to intensification phase, which consisted of a maximum of three repetitive high‐dose cytarabine cycles (at a dose ranging between 2 and 4 g/sqm per day days 1–5 plus idarubicine 8 mg/m^2^ per day on day 1 and 2 or 10 mg/sqm day 1) followed or not by autologous PBSC rescue (maximum of three cycles), whereas high‐risk patients were addressed to HLA‐matched allo‐SCT.

Concerning our series of patients, after remission induction, 93/104 (83%) and 80/104 (77%) patients were addressed to consolidation with PBSC collection and intensification with at least one cycle of high‐dose cytarabine with autologous PBSC rescue, respectively. 33/104 (32%) patients were addressed to allo‐SCT (Table 1).

### Sequential MRD monitoring

MRD monitoring by LAIP‐MFC from BM and by WT1 Q‐PCR from both BM and PB were systematically performed at the following time‐points: after induction course, after the first consolidation course and after the first intensification course. At each time‐point, MRD evaluation was performed at the time of recovery from PB cytopenia (usually between day +30 and +45 from each chemotherapy cycle).

Different combinations of fluorescent‐labeled monoclonal antibodies from BD Biosciences (CD7/CD33/CD3/CD2/CD34/CD4/CD8/CD45;CD5/CD22/CD19/CD10/CD13/CD20/CD33/CD45;CD15/CD34/CD14/CD117/CD33/CD64/CD11b/CD45; and CD64/CD56/HLA‐DR/CD13/CD34/CD16/CD33/CD45) were used to identify LAIPs on erythrocyte‐lysed BM samples obtained at the time of diagnosis as previously reported 14. Appropriate isotype‐matched negative controls served to assess background fluorescence intensity. The monoclonal antibody combinations characterizing the LAIP were used for MRD detection, which was assessed by acquiring 250,000 events. At least 20 LAIP‐positive events have been considered to define MRD‐positive sample. At the time of relapse, the same combinations of antibodies were applied and 50,000 events were collected. The analysis was performed on a FACSCanto II cytometer and data were analyzed with the FACSDiva software (BD Biosciences, San Jose, CA). The most frequent observed aberrations were as follows: asynchronous antigen expression (n: 48; 34%), lack of antigen expression (n: 36; 25%), lineage infidelity (n: 34; 24%), and overexpression (n: 25; 17%). LAIPs most frequently obtained were CD33+ CD4+ (13%) and CD33+ CD13− (13%). Immunophenotypic shift of the leukemic clone at relapse was observed in one case only.

Bone marrow and PB quantitative assessment of WT1 molecular levels was performed by Q‐PCR according to the ELN method as previously published 15, 16.

### Statistical analysis

Survival distributions (relapse‐free survival – RFS) were estimated using the Kaplan–Meier method 17. RFS was calculated from the date of 1st remission until the date of relapse or death, whichever occurred first. Transplanted patients were censored at the time of SCT. Differences in RFS were evaluated by log‐rank test. Cox proportional hazard regression model was used for univariate and multivariate analysis of factors associated with RFS. The following variables were analyzed for all patients: age, sex, FAB subtype, WBC, ELN risk category, unfavorable cytogenetics, morphological/cytogenetic remission after induction and biological parameters (LAIP, FLT3‐ITD, FLT3‐TKD, NPM1 mutations, and WT1 expression) at the time‐points previously indicated. Continuous variables were categorized as follows: each variable was first divided into four categories at approximately the 25th, 50th, and 75th percentile. If the hazard ratios (HRs) in 2 or more adjacent categories were not substantially different, these categories were grouped together. If no clear pattern was observed, the median was taken as the cut point. All *P* values were two‐sided and *P* < 0.05 was considered statistically significant.

## Results

### Risk stratification at diagnosis

The patients were stratified at diagnosis according to the ELN risk criteria 1. Neither by univariate nor by multivariate analysis did ELN risk impact on RFS. Other baseline clinical (age and sex), and biological variables (WBC count, cytogenetic alone, PB and BM‐WT1 level, NPM1 mutation and Flt3‐ITD/TKD mutation) were included in univariate and multivariate analysis, but only a WBC count greater than 58.500/mmc was independently associated with adverse RFS (HR 4.0; 95% CI 1.4–11.7; *P* = 0.01).

The median level of PB and BM WT1 at diagnosis was 1747 and 3621 × 10^4^ ABL copies, respectively. By univariate analysis on RFS, at least in our cohort of patients, none of the two values was able to dissect patients at different risk of relapse (HR 1.4 [95% CI 0.4–5.2] – *P* = 0.62 and HR 0.9 [95% CI 0.3–2.3] – *P* = 0.79, for PB and BM, respectively).

No significant association between a peculiar LAIP or BM/PB WT1 overexpression and any baseline clinical–pathological characteristic of the patients was found.

### Evaluation of predictive impact of postremission sequential MRD monitoring

Postremission predictive impact of MRD monitoring by LAIP‐MFC and by WT1 Q‐PCR on RFS was evaluated on the basis of MRD results assessed after induction, consolidation, and intensification, as previously stated.

By univariate analysis, BM‐WT1 ≥ 295 × 10^4^ ABL copies (HR 7.8; 95% CI 3.7–16.5 – *P* < 0.0001) and nonresponse (NR) to the first induction course were significantly associated with poor prognosis (HR 3.3; 95% CI 1.4–7.8; *P* = 0.005). After the 1st consolidation cycle, BM‐WT1 ≥ 121 × 10^4^ ABL copies (HR 5.2; 95% CI 2.4–11.4 – *P* < 0.0001), PB‐WT1 ≥ 18 × 10^4^ ABL copies (HR 7.9; 95% CI 3.6–17.4 – *P* < 0.0001), LAIP ≥ 0.2% (HR 3.3; 95% CI 1.6–7.1 – *P* = 0.001) were associated with higher incidence of relapse. After 1st intensification, BM‐WT1 ≥ 150 × 10^4^ ABL copies (HR 7.8; 95% CI 3.1–19.4 – *P* < 0.0001), PB‐WT1 ≥ 16 × 10^4^ ABL copies (HR 12.2; 95% CI 4.4–33.2 – *P* < 0.0001), a LAIP ≥ 0.27% (HR 4.6; 95% CI 1.9–10.8 – *P* = 0.0006) significantly impaired the RFS (Table 2A).

**Table 2:** Predictive impact of postremission sequential MRD‐monitoring: results of (a) univariate analysis^a^ and multivariate anaylsis^a^

	Time‐point					
	Postinduction		Post 1st consolidation		Post 1st intensification	
Variable^a^	HR (95% CI)	*P*	HR (95% CI)	*P*	HR (95% CI)	*P*
(A) Results of univariate analysis						
No response after induction	3.3 (1.4–7.8)	0.005	–	–	–	–
BM‐WT1 ≥ 295 × 10^4^ ABL copies	7.8 (3.7–16.5)	<0.0001	–	–	–	–
BM‐WT1 ≥ 121 × 10^4^ ABL copies	–	–	5.2 (2.4–11.4)	<0.0001	–	–
PB‐WT1 ≥ 18 × 10^4^ ABL copies	–	–	7.9 (3.6–17.4)	<0.0001	–	–
LAIP ≥ 0.2%	–	–	3.3 (1.6–7.1)	0.001	–	–
BM‐WT1 ≥ 150 × 10^4^ ABL copies	–	–	–	–	7.8 (3.1–19.4)	<0.0001
PB‐WT1 ≥ 16 × 10^4^ ABL copies	–	–	–	–	12.2 (4.4–33.2)	<0.0001
LAIP ≥ 0.27%	–	–	–	–	4.6 (1.9–10.8)	0.0006
(B) Results of multivariate anaylsis^a^						
BM‐WT1 ≥ 121 × 10^4^ ABL copies	–	–	4.1 (1.3–13.1)	0.02	–	–
LAIP ≥ 0.2%	–	–	3.3 (1.5–7.0)	0.0001	–	–
PB‐WT1 ≥ 16 × 10^4^ ABL copies	–	–	–	–	10.2 (3.2–32.1)	0.0001

a Significant variables only are reported.

**Figure 1:**
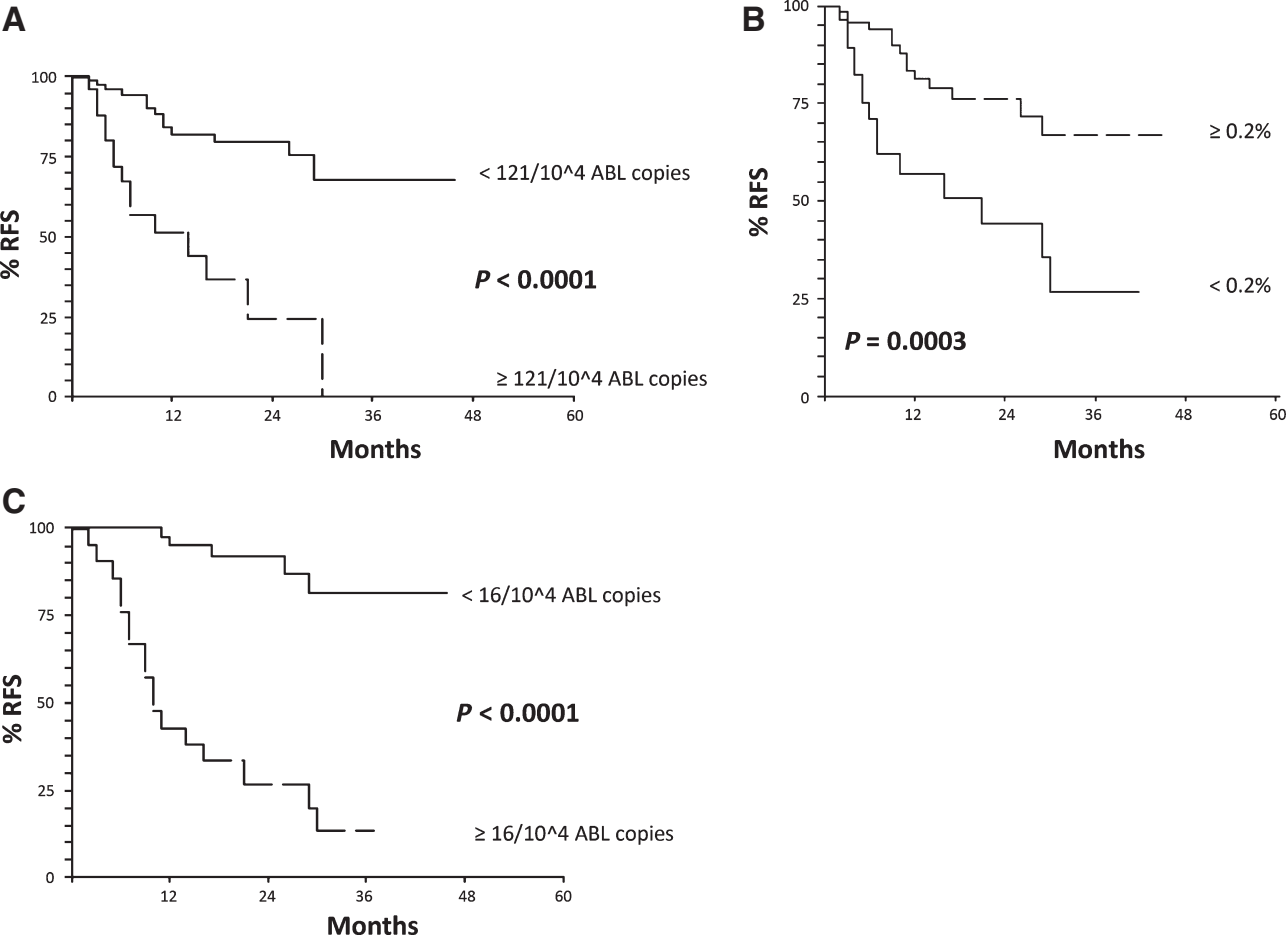
(A) Relapse‐free survival (RFS) of the patients according to BM‐WT1 after 1st consolidation. At the median follow up (12.5 months): RFS 82% (95% CI 72–92) for BM‐WT1 < 121/10^4^ ABL copies versus 51% (95% CI 30–72) for BM‐WT1 ≥ 121/10^4^ ABL copies (assessment after 1st consolidation). (B) RFS of the patients according to Leukemia Associated Immunophenotype (LAIP) after 1st consolidation. At the median follow up (12.5 months): RFS 81% (95% CI 70–92) for LAIP ≥ 0.2% versus 57% (95% CI 37–77) for LAIP decrease <0.2% (assessment after 1st consolidation). (C) RFS of the patients according to PB‐WT1 after intensification (1st cycle). At the median follow up (12.5 months): RFS 95% (95% CI 88–100) for PB‐WT1 < 16/10^4^ ABL copies versus 43% (95% CI 22–64) for PB‐WT1 ≥ 16/10^4^ ABL copies (assessment after postintensification – 1st cycle).

The results of multivariate analysis are reported in Table 2B. After 1st consolidation, BM‐WT1 ≥ 121 × 10^4^ ABL copies (HR 4.1; 95% CI 1.3–13.1 – *P* = 0.02) and LAIP ≥ 0.2% (HR 3.3; 95% CI 1.5–7.0 – *P* = 0.0001) were independently associated with adverse RFS. At the median follow up, the RFS of patients with BM‐WT1 < versus ≥ 121 × 10^4^ ABL copies was 82% (95% CI 72‐92) versus 51% (95% CI 30–72) (*P* < 0.0001, Fig. 1A). Similarly, the RFS of patients with LAIP ≥ 0.2% versus <0.2% was 81% (95% CI 70–92) versus 57% (95% CI 37–77), respectively (*P* = 0.0003, Fig. 1B). After 1st intensification, PB‐WT1 ≥ 16 × 10^4^ ABL copies (HR 10.2; 95% CI 3.2–32.1 – *P* = 0.0001) was the only independent predictive factor for adverse long‐term outcome. As a consequence, the RFS of patients with PB‐WT1 < versus ≥ 16 × 10^4^ ABL copies was 95% (95% CI 88–100) versus 43% (95% CI 22–64) (*P* < 0.0001, Fig. 1C). The clinical and biological characteristics of the patients with BM WT1 > × ≥ 121 × 10^4^/10^4^ ABL and LAIP ≥/< 0.2% after 1st consolidation, as well as those of the patients with PB‐WT1 < / ≥ 16 × 10^4^ ABL copies after 1st intensification were well balanced and no statistically significant differences were observed (see Table S1A, B and C).

Univariate and multivariate analysis was also performed within each of the different ELN risk categories. By multivariate analysis, BM‐WT1 ≥ 121 × 10^4^ ABL copies after the 1st consolidation cycle significantly dissected the patients at high risk of relapse both in the ELN Int‐1 ([HR 14.4; 95% CI 2–119; *P* = 0.01] and Int‐2 risk category [HR 11.7; 95% CI 1–125]; *P* = 0.04). Similarly, LAIP ≥ 0.2% after the 1st consolidation cycle significantly identified the patients at high risk of relapse in the ELN Int‐2 risk group only (HR 10.1; 95% CI 1–94; *P* = 0.04).

### Incidence of morphological relapse in MRD‐positive patients

According to the baseline characteristics, first‐line allo‐SCT was planned as an intensification treatment program in ELN high‐risk patients. Looking at these patients, they represented 20/104 (19%); of these 4 (20%) relapsed and 18 (90%) were allotransplanted in the first line. Since allo‐SCT was mandatory for these categories of patients, we excluded them from subsequent analysis.

In ELN favorable‐risk group, 10/37 (27%) patients relapsed and 1/37 (3%) was transplanted in first remission (molecular relapse in NPM1‐positive case); 3/37 (8%) were transplanted in 2nd CR (Table 3). According to MRD positivity assessed after consolidation and after intensification we observed that 3/12 (25%) and 4/5 (80%) MRD‐positive cases relapsed, respectively. The incidence of relapse rose to 100% in the two cases with MRD positivity both after consolidation and after intensification (Table 3).

**Table 3:** Postremission risk‐adapted therapy: transplanted versus transplantable patients

			Transplanted patients		Relapses in MRD‐positive patients			Transplantable patients before disease relapse according to MRD positivity		
ELN‐risk category	*N*°	Relapses	Allo‐SCT 1st CR	Allo‐SCT 2nd CR	Postconsolidation	Postintensification	Both	Postconsolidation	Postintensification	Both
Favorable	37	10/37 (27%)	1/37 (3%)	3/37 (8%)	3/12 (25%)^a^	4/5 (80%)^a^	2/2 (100%)^a^	2/12 (16%)^a^	3/5 (60%)^a^	1/2 (50%)^a^
Intermediate 1 – 2	47	16/47 (34%)	7/47 (15%)	7/47 (15%)	11/20 (55%)	11/15 (73%)	9/11 (82%)	11/20 (55%)	11/15 (73%)	9/11 (82%)
Overall	84	36/84 (43%)	8/84 (9%)	10/84 (12%)	14/32 (43%)	15/20 (75%)	11/13 (85%)	13/32 (41%)	14/20 (70%)	10/13 (77%)

a 1 patient with MRD positivity after consolidation, 1 patient with MRD positivity after intensification and 1 patient with MRD positivity both after consolidation and intensification who relapsed were over 70 years of age and thus not eligible for allo‐SCT.

Within the ELN intermediate 1–2 risk categories, allo‐SCT has been offered front‐line in selected patients such as those primary refractory or with disease non‐responsive to the 1st induction course or with high‐risk molecular markers (e.g., Flt3‐ITD without NPM1 mutation). ELN Intermediate 1–2 risk patients accounted for 47/104 (37%); 16/47 (34%) relapsed; 7/47 (15%) were transplanted in first remission (3 Flt3‐ITD positive, 2 AML with extramedullary leukemia, 2 1st induction failure); 7/47 (15%) were transplanted after relapse in 2nd CR (Table 3). On the basis of MRD positivity (either WT1 Q‐PCR or LAIP‐MFC) measured after consolidation and after intensification, we observed that 11/20 (55%) and 11/15 (73%) MRD‐positive cases relapsed, respectively. Moreover, 9/11 (82%) cases with MRD positivity both after consolidation and intensification relapsed (Table 3).

## Discussion

In the absence of new and more effective antileukemic drugs, the improvement of outcome observed in adult AML patients during the last decades has been mainly due to efforts in optimizing postremission therapy 2, 3. Two major factors played a role concerning this point: the progressively better identification of patients at high risk of relapse 1, 18 and, in particular, the more extensive use of allo‐SCT in 1st CR in patients selected on the basis of cytogenetic and molecular high‐risk markers 4. This last category of patients accounts for 40–50% of cases. Therefore, approximately half of AML patients, who are included in the ELN low/intermediate risk categories, are generally excluded from first‐line allo‐SCT intensification procedures in order to avoid the risk of high transplant‐related mortality.

However, both low and intermediate risk patients at diagnosis may eventually relapse in 20–40% of cases and they should be retreated before being transplanted in 2nd CR, with more resistant disease and suboptimal clinical conditions. In our cohort of low and intermediate‐risk AML patients relapses occurred in 27% and 34% of cases, respectively, and only a minority of these patients were actually allotransplanted in 2nd CR (Table 3). In view of postremission therapy optimization, the evaluation of MRD during and after treatment may be a powerful and accurate tool to improve risk assignment, as initially established in many hematological diseases 19, 20 With respect to other diseases, AMLs almost represent an exception, even if studies of other groups clearly indicate that the combination of prognostic parameters detected before treatment (cytogenetic/molecular) and during treatment (CR after one or two cycles) may improve outcome prediction 6 and that MRD monitoring, as an expression of disease debulking and drug sensitivity, may be extremely important to guide postremission risk‐adapted treatment 7, 9, 10, 11, 12, 21, 22, 23, 24, 25, 26.

In this study, we firstly performed a risk stratification of our patients according to the baseline clinical and biological characteristics. None of the studied variables but a WBC count greater than 58.500/mmc was independently associated with adverse RFS (HR 4.0 by multivariate analysis; 95% CI 1.4–11.7; *P* = 0.01). The result of this analysis may be influenced by the intensive therapeutic program adopted in our series, which may have hampered the prognostic significance of some commonly accepted outcome predictors such as cytogenetics. We then focused on the longitudinal monitoring of MRD with LAIP‐MFC and WT1 Q‐PCR on PB and BM simultaneously, with the aim of evaluating their usefulness in the prediction of relapse, comparing the efficiency of the two methods and evaluating the most accurately predictive time‐point for relapse. Multivariate analysis clearly identified BM‐WT1 ≥ 121 × 10^4^ ABL copies (HR 4.1; *P* = 0.02) and LAIP ≥ 0.2% (HR 3.3; *P* = 0.0001) after 1st consolidation, and PB‐WT1 ≥ 16 × 10^4^ ABL copies (HR 10.2; *P* = 0.0001) after 1st intensification as associated with dismal prognosis (Table 2, Fig. 1).

A potential prospective use of these MRD‐positive values to plan postremission risk‐adapted therapy has been suggested in Table 3. Based on the predictive value of MRD monitoring on relapse, 5/37 (19%) ELN favorable‐risk patients could have been considered for allotransplant in 1st CR, facing a relapse risk of 25% and 80%, respectively, if MRD positivity was detected after consolidation or intensification. Similarly, 22/47 (47%) ELN intermediate‐risk patients could have been considered for allotransplant in 1st CR, facing a relapse risk of 55% and 73%, respectively, if MRD positivity was detected after consolidation or intensification. Finally, in both ELN groups the persistence of MRD positivity after consolidation and intensification was predictive of relapse in at least 80% of cases, thus supporting the indication of an allotransplant. We are aware that the number of patients in each group is relatively small and that any conclusion should be drawn carefully, but we think that this approach could be helpful to clinicians and patients, in order to better evaluate the balance between the risk of relapse and that of an allotransplant, the latter being of benefit when the risk of leukemia relapse exceeds 35–40% 27.

MFC on LAIP and WT1 Q‐PCR techniques became very popular for detecting leukemic cells at submicroscopic level, mainly because they offered the opportunity of a MRD longitudinal measure 28. MFC shows the advantage of being available in almost all hematological‐oriented laboratories, it is relatively easy but is operator‐dependent, is not completely standardized and the immunophenotypic shift of the leukemic clone may hamper its power in predicting relapse. On the other hand, Q‐PCR on target genes is easy, highly standardized and relatively cheap, but it is applicable mainly in AML patients with known molecular aberrations (e.g., molecular rearrangements arising from chromosomal translocations such as in CBF leukemias, or gene mutations such as NPM1 and Flt3‐ITD), which are observed in a minority of cases 29, 30, 31. Nevertheless, the possibility to quantify with Q‐PCR the WT1 gene, which is overexpressed in up to 80–90% of AML cases, overcomes this limit and offers the opportunity to monitor a MRD marker in the great majority of patients, although its specificity in detecting the leukemic clone remains controversial. We used both the LAIP‐MFC and WT1 Q‐PCR in order to evaluate the efficiency of the two methods and we decided to monitor the MRD at a very early time‐point (after induction), an intermediate time‐point (after consolidation), and a late time‐point (at the end of the intensification program). In the end, we cannot say that one is clearly better than the other and we have seen that both methods are useful to stratify patients at high risk of relapse as similarly reported by other Authors 32, 33, 34. These results have been confirmed by other groups, who reported the power of WT1 monitoring in detecting patients at high risk of relapse, but no conclusive data are available concerning the cut‐off for positive versus negative samples, as well as the optimal time‐point for its assessment 23, 36, 37, 38, 39, 40, 41, 42, 43. Concerning this latter point, data coming from the published studies are not conclusive. In particular, a very early time‐point for WT1 monitoring (postinduction) 15, 36, 37, 38, 39, 41, 42, 43, but also a later time‐point (postconsolidation or pre‐allo‐SCT) 38, 41, 42, 43 have been reported to significantly predict the outcome. Concerning LAIP‐MFC sensitivity and efficiency, our data are concordant with those reported by the GIMEMA group and suggest that the most accurate predictive time‐point of MRD assessment is probably after consolidation 14, 44. In particular, we have seen that, within favorable and intermediate‐risk groups, MRD positivity after consolidation predicts relapse in about 40% of MRD‐positive AML patients, but the accuracy of relapse prediction increases to 75% when the evaluation of MRD positivity is made after intensification. On other hand, other Groups observed that LAIP‐MFC positivity detected at an earlier time‐point (after induction) is significantly associated with increased relapse risk 25, 45, 46.

According to our experience and to the data reported in literature, MRD monitoring has to be considered dynamically: the more we advance in the treatment program, the more the level of MRD positivity is reduced, but at the same time, the greater becomes the accuracy of the predictive power of MRD positivity on relapse. Only taking into account the dynamic nature of this phenomenon, we can explain the discordance of the cut‐off values for positive versus negative samples observed by different groups. Also in our experience, for example, the predictive value of PB‐WT1 ≥ 16 × 10^4^ ABL copies measured after 1st intensification is greater than PB‐WT1 ≥ 5 × 10^4^ ABL copies measured before transplantation after completion of the treatment program, as already published on a selected cohort of the same patients 16.

As both LAIP and WT1 monitoring proved to be effective and highly concordant in relapse prediction, any of them could be chosen in relationship with the expertise and the facilities available in different centers. In the meantime, we think the most important effort is to make a good choice in terms of the method used for MRD monitoring and good standardization and close monitoring of MRD. As suggested by our data, the accuracy of indication to allo‐SCT depends on when we detect MDR positivity and on the number of time‐points (single or multiple) used for this detection. Moreover, MDR positivity as an indicator to allo‐SCT intensification should be combined with other clinical and biological factors detected at disease onset. Only through this integrated system of evaluation we think that the treatment program could be optimized and customized for each patient.

## Conflict of Interest

None declared.

## Supporting information


**Table S1.** (A) Clinical and biological features of patients with BM WT1 ≥ 121 × 10^4^ ABL copies versus BM WT1 < 121 × 10^4^ ABL copies after 1st consolidation. (B) Clinical and biological features of patients with LAIP ≥ 0.2% versus LAIP < 0.2% after 1st consolidation. (C) Clinical and biological features of patients with PB WT1 ≥ 16 × 10^4^ ABL copies versus PB WT1 < 16 × 10^4^ ABL copies after 1st intensification.

## References

[1] Döhner, H. , E. H. Estey , S. Amadori , F. R. Appelbaum , T. Büchner , A. K. Burnett , et al. 2010 Diagnosis and management of acute myeloid leukemia in adults: recommendations from an international expert panel, on behalf of the European LeukemiaNet. Blood 115:453–474. 19880497 10.1182/blood-2009-07-235358

[2] Burnett, A. K . 2012 Treatment of acute myeloid leukemia: are we making progress? Hematology 2012:1–6. 23233553 10.1182/asheducation-2012.1.1

[3] Schlenk, R. F. 2014 Post‐remission therapy for acute myeloid leukemia. Haematologica 99:1663–1670. 25420282 10.3324/haematol.2014.114611 PMC4222459

[4] Schiller, G. J . 2013 High‐risk acute myelogenous leukemia: treatment today … and tomorrow. Hematology 2013:201–208. 24319182 10.1182/asheducation-2013.1.201

[5] Schlenk, R. F. , K. Döhner , J. Krauter , S. Fröhling , A. Corbacioglu , L. Bullinger , et al. 2008 Mutations and treatment outcome in cytogenetically normal acute myeloid leukemia. N. Engl. J. Med. 358:1909–1918. 18450602 10.1056/NEJMoa074306

[6] Wheatley, K. , A. K. Burnett , A. H. Goldstone , R. G. Gray , I. M. Hann , C. J. Harrison , et al. 1999 A simple, robust, validated and highly predictive index for the determination of risk‐directed therapy in acute myeloid leukaemia derived from the MRC AML 10 trial. United Kingdom Medical Research Council's Adult and Childhood Leukaemia Working Parties. Br. J. Haematol. 107:69–79. 10520026 10.1046/j.1365-2141.1999.01684.x

[7] Buccisano, F. , L. Maurillo , A. Spagnoli , M. I. Del Principe , E. Ceresoli , F. Lo Coco , et al. 2009 Monitoring of minimal residual disease in acute myeloid leukemia. Curr. Opin. Oncol. 21:582–588. 19667983 10.1097/CCO.0b013e3283311856

[8] Buccisano, F. , L. Maurillo , A. Spagnoli , M. I. Del Principe , D. Fraboni , P. Panetta , et al. 2010 Cytogenetic and molecular diagnostic characterization combined to postconsolidation minimal residual disease assessment by flow cytometry improves risk stratification in adult acute myeloid leukemia. Blood 116:2295–2303. 20548095 10.1182/blood-2009-12-258178

[9] Buccisano, F. , L. Maurillo , M. I. Del Principe , G. Del Poeta , G. Sconocchia , F. Lo‐Coco , et al. 2012 Prognostic and therapeutic implications of minimal residual disease detection in acute myeloid leukemia. Blood 119:332–341. 22039260 10.1182/blood-2011-08-363291

[10] Maurillo, L. , F. Buccisano , A. Spagnoli , G. Del Poeta , P. Panetta , and B. Neri . 2007 Monitoring of minimal residual disease in adult acute myeloid leukemia using peripheral blood as an alternative source to bone marrow. Haematologica 92:605–611. 17488683 10.3324/haematol.10432

[11] Maurillo, L. , F. Buccisano , M. I. Del Principe , G. Del Poeta , A. Spagnoli , and P. Panetta . 2008 Toward optimization of postremission therapy for residual disease‐positive patients with acute myeloid leukemia. J. Clin. Oncol. 26:4944–4951. 18606980 10.1200/JCO.2007.15.9814

[12] Maurillo, L. , F. Buccisano , A. Piciocchi , M. I. Del Principe , C. Sarlo , and A. Di Veroli . 2015 Minimal residual disease as biomarker for optimal biologic dosing of ARA‐C in patients with acute myeloid leukemia. Am. J. Hematol. 90:125–131. 25377359 10.1002/ajh.23893

[13] Intermesoli, T. , E. Oldani , G. Rossi , E. Pogliani , F. Marmont , I. Cavattoni , et al. 2010 Two‐step response‐oriented induction predicts long‐term outcome of adult patients with standard‐ and high‐risk acute myeloid leukaemia (AML): a Northern Italy Leukaemia Group (NILG) study. Haematologica 95(suppl. 2):26, abs. 0065

[14] Venditti, A. , F. Buccisano , G. Del Poeta , L. Maurillo , A. Tamburini , C. Cox , et al. 2000 Level of minimal residual disease after consolidation therapy predicts outcome in acute myeloid leukemia. Blood 96:3948–3952. 11090082

[15] Cilloni, D. , A. Renneville , F. Hermitte , R. K. Hills , S. Daly , and J. V. Jovanovic . 2009 Real‐time quantitative polymerase chain reaction detection of minimal residual disease by standardized WT1 assay to enhance risk stratification in acute myeloid leukemia: a European LeukemiaNet study. J. Clin. Oncol. 27:5195–5201. 19752335 10.1200/JCO.2009.22.4865

[16] Malagola, M. , C. Skert , G. Ruggeri , A. Turra , R. Ribolla , V Cancelli , et al. 2014 Peripheral blood WT1 expression predicts relapse in AML patients undergoing allogeneic stem cell transplantation. Biomed. Res. Int. 2014:1–5. 10.1155/2014/123079 PMC4150519 25202702

[17] Kaplan, E. R. , and P. Mejer . 1958 Non parametric estimation from incomplete observation. J. Am. Stat. Assoc. 53:457–481.

[18] Döhner, K. , and P. Paschka . 2007 Intermediate‐risk acute myeloid leukemia therapy: current and future. Hematology 2014:34–43. 25696832 10.1182/asheducation-2014.1.34

[19] Pott, C. 2011 Minimal residual disease detection in mantle cell lymphoma: technical aspects and clinical relevance. Semin. Hematol. 48:172–184. 21782059 10.1053/j.seminhematol.2011.05.002

[20] van Dongen, J. J. , van der Velden V. H. , M. Brüggemann , and A. Orfao . 2015 Minimal residual disease (MRD) diagnostics in acute lymphoblastic leukemia (ALL): need for sensitive, fast and standardized technologies. Blood 125:3996–4009. 25999452 10.1182/blood-2015-03-580027 PMC4490298

[21] Bastos‐Oreiro, M. , A. Perez‐Corral , C. Martínez‐Laperche , L. Bento , C. Pascual , and M. Kwon . 2014 Prognostic impact of minimal residual disease analysis by flow cytometry in patients with acute myeloid leukemia before and after allogeneic hemopoietic stem cell transplantation. Eur. J. Haematol. 93:239–246. 24702162 10.1111/ejh.12336

[22] Chen, X. , H. Xie , B. L. Wood , R. B. Walter , J. M. Pagel , P. S. Becker, et al. 2015 Relation of clinical response and minimal residual disease and their prognostic impact on outcome in acute myeloid leukemia. J. Clin. Oncol. 33:1258–1264. 25732155 10.1200/JCO.2014.58.3518

[23] Cilloni, D. , E. Gottardi , D. De Micheli , A. Serra , G. Volpe , F. Messa , et al. 2002 Quantitative assessment of WT1 expression by real time quantitative PCR may be a useful tool for monitoring minimal residual disease in acute leukemia patients. Leukemia 16:2115–2121. 12357365 10.1038/sj.leu.2402675

[24] Kern, W. , D. Voskova , C. Schoch , W. Hiddemann , S. Schnittger , and T. Haferlach . 2004 Determination of relapse risk based on assessment of minimal residual disease during complete remission by multiparameter flow cytometry in unselected patients with acute myeloid leukemia. Blood 104:3078–3085. 15284114 10.1182/blood-2004-03-1036

[25] San Miguel, J. F. , A. Martínez , A. Macedo , M. B. Vidriales , C. López‐Berges , and M. González . 1997 Immunophenotyping investigation of minimal residual disease is a useful approach for predicting relapse in acute myeloid leukemia patients. Blood 90:2465–2470. 9310499

[26] Terwijn, M. , W. L. van Putten , A. Kelder , V. H. van der Velden , R. A. Brooimans , and T. Pabst . 2013 High prognostic impact of flow cytometric minimal residual disease detection in acute myeloid leukemia: data from the HOVON/SAKK AML 42A study. J. Clin. Oncol. 31:3889–3897. 24062400 10.1200/JCO.2012.45.9628

[27] Cornelissen, J. J. , W. L. van Putten , L. F. Verdonck , M. Theobald , E. Jacky , and S. M. Daenen . 2007 Results of a HOVON/SAKK donor versus no‐donor analysis of myeloablative HLA‐identical sibling stem cell transplantation in first remission acute myeloid leukemia in young and middle‐aged adults: benefits for whom? Blood 109:3658–3666. 17213292 10.1182/blood-2006-06-025627

[28] Kayser, S. , R. B. Walter , W. Stock , and R. F. Schlenk . 2015 Minimal residual disease in acute myeloid leukemia‐current status and future perspectives. Curr. Hematol. Malig. Rep. 10:132–144. 25994952 10.1007/s11899-015-0260-7

[29] Yin, J. A. , M. A. O'Brien , R. K. Hills , S. B. Daly , K. Wheatley , and A. K. Burnett . 2012 Minimal residual disease monitoring by quantitative RT‐PCR in core binding factor AML allows risk stratification and predicts relapse: results of the United Kingdom MRC AML‐15 trial. Blood 120:2826–2835. 22875911 10.1182/blood-2012-06-435669

[30] Schnittger, S. , W. Kern , C. Tschulik , T. Weiss , F. Dicker , and B. Falini . 2009 Minimal residual disease levels assessed by NPM1 mutation‐specific RQ‐PCR provide important prognostic information in AML. Blood 114:2220–2231. 19587375 10.1182/blood-2009-03-213389

[31] Abdelhamid, E. , C. Preudhomme , N. Helevaut , O. Nibourel , C. Gardin , and P. Rousselot . 2012 Minimal residual disease monitoring based on FLT3 internal tandem duplication in adult acute myeloid leukemia. Leuk. Res. 36:316–323. 22129478 10.1016/j.leukres.2011.11.002

[32] Marani, C. , M. Clavio , R. Grasso , N. Colombo , F. Guolo , and A. Kunkl . 2013 Integrating post induction WT1 quantification and flow‐cytometry results improves minimal residual disease stratification in acute myeloid leukemia. Leuk. Res. 37:1606–1611. 23891447 10.1016/j.leukres.2013.07.005

[33] Rossi, G. , M. M. Minervini , A. M. Carella , C. de Waure , F. di Nardo , and L. Melillo . 2012 Comparison between multiparameter flow cytometry and WT1‐RNA quantification in monitoring minimal residual disease in acute myeloid leukemia without specific molecular targets. Leuk. Res. 36:401–406. 22196957 10.1016/j.leukres.2011.11.020

[34] Rossi, G. , M. M. Minervini , L. Melillo , F. di Nardo , C. de Waure , and P. R. Scalzulli . 2014 Predictive role of minimal residual disease and log clearance in acute myeloid leukemia: a comparison between multiparameter flow cytometry and Wilm's tumor 1 levels. Ann. Hematol. 93:1149–1157. 24554303 10.1007/s00277-014-2029-9

[35] Cilloni, D. , F. Messa , F. Arruga , I. Defilippi , E. Gottardi , and M. Fava . 2008 Early prediction of treatment outcome in acute myeloid leukemia by measurement of WT1 transcript levels in peripheral blood samples collected after chemotherapy. Haematologica 93:921–924. 18443273 10.3324/haematol.12165

[36] Cilloni, D. 2009 Is WT1 helping the molecular monitoring of minimal residual disease to get easier in acute myeloid leukaemia? Leuk. Res. 33:603–604. 19269525 10.1016/j.leukres.2008.11.002

[37] Nomdedéu, J. F. , M. Hoyos , M. Carricondo , E. Bussaglia , C. Estivill , and J. Esteve . 2013 Bone marrow WT1 levels at diagnosis, post‐induction and post‐intensification in adult de novo AML. Leukemia 27:157–164. 10.1038/leu.2013.111 23584566

[38] Nowakowska‐Kopera, A. , T. Sacha , I. Florek , M. Zawada , S. Czekalska , and A. B. Skotnicki . 2009 Wilms’ tumor gene 1 expression analysis by real‐time quantitative polymerase chain reaction for monitoring of minimal residual disease in acute leukemia. Leuk. Lymphoma 50:1326–1332. 19811333 10.1080/10428190903050021

[39] Østergaard, M. , L. H. Olesen , H. Hasle , E. Kjeldsen , and P. Hokland . 2004 WT1 gene expression: an excellent tool for monitoring minimal residual disease in 70% of acute myeloid leukaemia patients ‐ results from a single‐centre study. Br. J. Haematol. 125:590–600. 15147374 10.1111/j.1365-2141.2004.04952.x

[40] Qin, Y. , H. Zhu , B. Jiang , J. Li , X. Lu , and L. Li . 2009 Expression patterns of WT1 and PRAME in acute myeloid leukemia patients and their usefulness for monitoring minimal residual disease. Leuk. Res. 33:384–390. 18950857 10.1016/j.leukres.2008.08.026

[41] Shibasaki, Y. , Y. Seki , T. Tanaka , S. Miyakoshi , K. Fuse , and T. Kozakai . 2015 The association of level of reduction of Wilms’ tumor gene 1 mRNA transcript in bone marrow and outcome in acute myeloid leukemia patients. Leuk. Res. 39:667–671. 25890432 10.1016/j.leukres.2015.03.021

[42] Weisser, M. , W. Kern , S. Rauhut , C. Schoch , W. Hiddemann , and T. Haferlach . 2005 Prognostic impact of RT‐PCR‐based quantification of WT1 gene expression during MRD monitoring of acute myeloid leukemia. Leukemia 19:1416–1423. 15920493 10.1038/sj.leu.2403809

[43] Yoon, J. H. , H. J. Kim , S. H. Shin , S. A. Yahng , S. E. Lee , and B. S. Cho . 2013 Serial measurement of WT1 expression and decrement ratio until hematopoietic cell transplantation as a marker of residual disease in patients with cytogenetically normal acute myelogenous leukemia. Biol. Blood Marrow Transplant. 19:958–966. 23542687 10.1016/j.bbmt.2013.03.013

[44] Buccisano, F. , L. Maurillo , V. Gattei , G. Del Poeta , M. I. Del Principe , and M. C. Cox . 2006 The kinetics of reduction of minimal residual disease impacts on duration of response and survival of patients with acute myeloid leukemia. Leukemia 20:1783–1789. 16838027 10.1038/sj.leu.2404313

[45] Freeman, S. D. , P. Virgo , S. Couzens , D. Grimwade , N. Russell , and R. K. Hills . 2013 Prognostic relevance of treatment response measured by flow cytometric residual disease detection in older patients with acute myeloid leukemia. J. Clin. Oncol. 31:4123–4131. 24062403 10.1200/JCO.2013.49.1753

[46] San Miguel, J. F. , M. B. Vidriales , C. López‐Berges , J. Díaz‐Mediavilla , N. Gutiérrez , and C. Cañizo . 2001 Early immunophenotypical evaluation of minimal residual disease in acute myeloid leukemia identifies different patient risk groups and may contribute to postinduction treatment stratification. Blood 98:1746–1751. 11535507 10.1182/blood.v98.6.1746

