# Supplementary material for: Postremission sequential monitoring of minimal residual disease by WT1 Q‐PCR and multiparametric flow cytometry assessment predicts relapse and may help to address risk‐adapted therapy in acute myeloid leukemia patients
Source: Cancer Med. 2015 Dec 29;5(2):265–74. doi: 10.1002/cam4.593 (PMC4735778; doi:10.1002/cam4.593)
Supplement: Supplementary file 1 — Table S1. (A) Clinical and biological features of patients with BM WT1 ≥ 121 × 104 ABL copies versus BM WT1 < 121 × 104 ABL copies after 1st consolidation. (B) Clinical and biological features of patients with LAIP ≥ 0.2% versus LAIP < 0.2% after 1st consolidation. (C) Clinical and biological features of patients with PB WT1 ≥ 16 × 104 ABL copies versus PB WT1 < 16 × 104 ABL copies after 1st intensification. [file CAM4-5-265-s001.doc]

**Table 1a. Clinical and biological features of patients with BM WT1 ≥ 121/10^4 ABL copies vs BM WT1 < 121/10^4 ABL copies after 1^st^ consolidation**

|  | **BM WT1 ≥ 121/10^4 ABL copies (n=25)**  **N (%)** | **BM WT1 < 121/10^4 ABL copies (n=75)**  **N (%)** | **P** |
| --- | --- | --- | --- |
| **Median age (range)** | 60 (18-74) | 56 (23-75) | 0,13 |
| **Sex** *(male)* | 17 (68) | 38 (51) | 0,13 |
| **Median WBC count / μl (range)** | 3570 (420-101600) | 10650 (400-218700) | 0,11 |
| **Unfavourable cytogenetic**** | 10 (40) | 30 (40) | 1 |
| **ELN risk category*** |  |  |  |
| *Favourable* | 6 (24) | 23 (31) | 0,27 |
| *Intermediate-1* | 9 (36) | 17 (23) | 0,19 |
| *Intermediate-2* | 5 (20) | 8 (11) | 0,23 |
| *High-risk* | 5 (20) | 11 (15) | 0,53 |
| **FLT3 ITD mutation** | 3 (12) | 15 (20) | 0,37 |
| **FLT3 TKD mutation** | 1 (4) | 5 (7) | 0,63 |
| **NPM1 mutation** | 8 (32) | 30 (40) | 0,47 |
| **NPM1/FLT3ITD mutations** | 2 (8) | 8 (11) | 0,83 |

**Table 1b. Clinical and biological features of patients with LAIP ≥ 0,2% vs LAIP < 0,2% after 1^st^ consolidation**

|  | **LAIP ≥ 0,2% (n=28)**  **N (%)** | **LAIP < 0,2% (n=72)**  **N (%)** | **P** |
| --- | --- | --- | --- |
| **Median age (range)** | 60 (18-74) | 57 (24-75) | 0,28 |
| **Sex** *(male)* | 18 (64) | 36 (50) | 0,20 |
| **Median WBC count / μl (range)** | 5875 (420-170000) | 9600 (400-218700) | 0,77 |
| **Unfavourable cytogenetic**** | 13 (46) | 27 (38) | 0,41 |
| **ELN risk category*** |  |  |  |
| *Favourable* | 8 (26) | 25 (35) | 0,56 |
| *Intermediate-1* | 10 (36) | 16 (22) | 0,17 |
| *Intermediate-2* | 5 (18) | 8 (11) | 0,37 |
| *High-risk* | 4 (14) | 12 (17) | 0,77 |
| **FLT3 ITD mutation** | 6 (21) | 12 (17) | 0,58 |
| **FLT3 TKD mutation** | 3 (11) | 3 (4) | 0,21 |
| **NPM1 mutation** | 8 (29) | 30 (42) | 0,22 |
| **NPM1/FLT3ITD mutations** | 3 (11) | 7 (10) | 0,88 |

**Table 1c. Clinical and biological features of patients with PB WT1 ≥ 16/10^4 ABL copies vs PB WT1 < 16/10^4 ABL copies after 1^st^ intensification.**

|  | **PB WT1 ≥ 16/10^4 ABL copies (n=21)**  **N (%)** | **PB WT1 < 16/10^4 ABL copies (n=61)**  **N (%)** | **P** |
| --- | --- | --- | --- |
| **Median age (range)** | 60 (18-75) | 55 (23-74) | 0,11 |
| **Sex** *(male)* | 13 (62) | 24 (39) | 0,08 |
| **Median WBC count / μl (range)** | 20000 (810-149000) | 4415 (400-218700) | 0,15 |
| **Unfavourable cytogenetic**** | 5 (24) | 17 (28) | 0,72 |
| **ELN risk category*** |  |  |  |
| *Favourable* | 5 (24) | 10 (16) | 0,45 |
| *Intermediate-1* | 8 (38) | 12 (20) | 0,09 |
| *Intermediate-2* | 3 (14) | 12 (20) | 0,58 |
| *High-risk* | 1 (5) | 5 (8) | 0,60 |
| **FLT3 ITD mutation** | 7 (33) | 12 (20) | 0,20 |
| **FLT3 TKD mutation** | 0 (0) | 1 (2) | 0,55 |
| **NPM1 mutation** | 6 (29) | 14 (23) | 0,60 |
| **NPM1/FLT3ITD mutations** | 6 (29) | 8 (13) | 0,10 |

**Table Legend:** LAIP: leukemia associated immunophenotype; ITD: internal tandem duplication; PBSC: peripheral blood stem cells.

* according to the ELN criteria (1)

** according to the ELN criteria (15)
